# Successful use of femoral nerve block with dexmedetomidine for fracture fixation of an intracapsular fracture of the femoral neck in a patient with severe aortic stenosis: a case report

**DOI:** 10.1186/s40981-017-0126-1

**Published:** 2017-10-03

**Authors:** Yui Yamane, Takeshi Omae, Keito Kou, Sonoko Sakuraba

**Affiliations:** Department of Anesthesiology and Pain Clinic, Juntendo University Shizuoka Hospital, Izunokuni, Shizuoka 1129 Japan

**Keywords:** Femoral nerve block, Aortic stenosis, Guideline

## Abstract

We described a case in which femoral nerve block (FNB) and lateral femoral cutaneous nerve block (LFCNB) with dexmedetomidine (DEX) was useful for open reduction and internal fixation (ORIF) of a femoral neck fracture in a patient with severe aortic stenosis. Cardiac surgery had been recommended but was declined by the patient. Thus, ORIF was selected because of the patient’s concomitant severe aortic stenosis. The anesthesia method used was FNB plus LFCNB with DEX, which achieved adequate local anesthesia. DEX was used to avoid respiratory depression because this patient has pulmonary hypertension. This patient had been sedative up to the end of surgery. Total operating time was 51 min, and the patient’s hemodynamics were stable throughout the perioperative period. There were no complications. In this case, anesthesia using a nerve block with DEX contributed to the safety of noncardiac surgery in a patient with severe cardiac disease under conservative treatment during the perioperative period.

## Background

In the emergency setting, we sometimes encounter the patients with coexisting severe comorbidities, especially cardiovascular disease. Emergency operation is a priority if cardiac surgery is recommended for these patients according to the 2014 American Heart Association/American College of Cardiology (AHA/ACC) guideline for management of patients with valvular heart disease [1]. In such cases, selection of the anesthesia method and monitoring is important for hemodynamic stability during the perioperative period. Here, we report a case in which femoral nerve block with dexmedetomidine (DEX) was useful for open reduction and internal fixation (ORIF) of a femoral neck fracture in a patient with severe aortic stenosis using the Flotrac/Vigileo system.

## Case presentation

A 68-year-old woman (body weight, 45 kg; height, 145 cm) fell while walking and visited our hospital with a chief complaint of hip pain. She had a history of hypertension, diabetes mellitus, aortic stenosis, atrial fibrillation, and schizophrenia. She lived in a care home, and detailed information was unavailable due to difficulty contacting her family. Previously, she had been hospitalized three times with a chief complaint of dyspnea. Cardiac surgery had been recommended but was declined by the patient.

On admission, her hemodynamics were stable, and there were no subjective or objective symptoms except for hip pain. After a detailed investigation, she was diagnosed as having intracapsular fracture of the femoral neck. Transthoracic echocardiography performed in the department of cardiovascular medicine showed an aortic valve area of 0.7 cm^2^ (aortic valve index, 0.49), aortic valve maximum pressure gradient of 96 mmHg, aortic regurgitation, and ejection fraction of 40%. And tricuspid regurgitation pressure gradient was 43 mmHg. Preoperative hemoglobin level was 8 g/dL. Based on these findings, she was diagnosed as having severe aortic stenosis.

Although bipolar hip arthroplasty was initially planned, ORIF was selected because of the patient’s concomitant severe aortic stenosis. The anesthesia method used was femoral nerve block with DEX. She was monitored using 5-lead electrocardiography and pulse oximetry. Continuous arterial pressure was measured after inserting a catheter into the radial artery. A PreSep Oximetry Catheter (Edwards Lifesciences, Irvine, CA, USA) was inserted via right jugular vein to measure central venous pressure (CVP) and central venous O_2_ saturation (ScvO_2_), and hemodynamic monitoring was performed using the FloTrac/Vigileo system (Edwards Lifesciences, Irvine, CA, USA). Hemodynamic values, including the cardiac index (CI), mean arterial pressure (MAP), heart rate (HR), CVP, systemic vascular resistance index (SVRI), stroke volume index (SVI), and ScvO_2_, were measured. Femoral nerve block was performed using a linear ultrasound probe (HFL50 15–6 MHz; SonoSite Inc., Bothell, WA, USA) and a 5-cm, 18-gauge Contiplex Tuohy needle (B-Braun Ltd., Tochigi, Japan) [2]. A 22-mL dose of ropivacaine 0.2% was injected around the femoral nerve, and the lateral femoral cutaneous nerve block (LFCNB) was achieved with a 10-mL dose of ropivacaine 0.2%. After obtaining adequate local anesthesia, DEX administration and surgery were started simultaneously. Sedative level was decided by the Observer’s Assessment of Alertness/Sedation (OAA/S) score. The dose of DEX was adjusted within the range of 0.2–0.7 μg/kg/h by using OAA/S score. Sedative level was 3 and PaCO_2_ was 39.3 mmHg at the conclusion of surgery. However, the patient showed signs of distress when operating on the femoral head, for which fentanyl 25 μg was administered four times. Total operating time was 51 min, and anesthesia time was 100 min. Total blood loss was 20 g, total fluid valance was + 230 mL, and wound length was 10 cm. The patient’s hemodynamics remained stable during the perioperative period (Table 1).

**Table 1:** Hemodynamic variables after FNB plus LFCNB

Variable		Time after FNB						
	Baseline (0 min)	10 min	20 min (start of DEX infusion)	30 min	40 min	50 min	60 min	End of surgery
MAP (mmHg)	78	78	80	77	77	78	76	77
HR (beat/min)	71	78	82	65	74	68	73	70
CI (L/min/m^2^)	2.5	2.6	2.5	2.6	2.6	2.5	2.6	2.5
SVI (mL/beat/m^2^)	35	33	30	40	35	37	36	36
CVP (mmHg)	5	5	5	6	5	5	5	5
SVRI (dyne sec m^2^/cm^5^)	2334	2244	2398	2182	2213	2334	2182	2302
ScvO_2_ (%)	70	69	71	71	72	71	71	71
PaCO_2_ (mmHg)	39.7		38.6		39.1		39.0	39.3

*FNB* femoral nerve block, *LFCNB* lateral femoral cutaneous nerve block, *DEX* dexmedetomidine, *MAP* mean arterial pressure, *HR* heart rate, *CI* cardiac index, *SVI* stroke volume index, *CVP* central venous pressure, *SVRI* systemic vascular resistance index, *ScvO*_*2*_ central venous O_2_ saturation, *PaCO*_*2*_ partial pressure of arterial carbon dioxide

After surgery, the patient was transferred to the intensive care unit while DEX was continued. Then, the dose of DEX was gradually tapered. Acetaminophen 1000 mg was administered for postoperative pain, after which there was no complaint of pain. She was transferred to the general ward a day after the surgery, and her hemodynamics remained stable throughout the perioperative period.

## Discussion

We described a case of anesthesia management for ORIF in a 68-year-old patient with various comorbidities, including severe aortic stenosis.

In this case, the patient was diagnosed as having severe aortic disease. According to the 2014 American Heart Association/American College of Cardiology (AHA/ACC) guideline for management of patients with valvular heart disease, aortic valve replacement is recommended as a class I treatment in patients with severe aortic stenosis [1]. ORIF was scheduled after obtaining informed consent and explaining the risks of untreated aortic stenosis. Severe aortic stenosis is associated with increased risk in noncardiac surgery, depending on the degree of aortic valve and left ventricular systolic function [1]. It was necessary to pay attention to fluid balance [1]. We used CVP to assess preload. Because this patient was spontaneously breathing, we could not use minimally invasive monitoring such as stroke volume variation.

We chose femoral nerve block plus DEX as the anesthetic method. We determined that hemodynamic stability was high on the patient’s list of priorities, because the psychiatric disorder was well controlled. In general, ORIF is performed under general or spinal-epidural anesthesia. Wood et al. examined the effects of anesthesia on intraoperative blood pressure and reported higher incidences of intraoperative hypotension with both general anesthesia (34.2%) and spinal-epidural anesthesia (29.7%) [3]. A femoral nerve block has a smaller effect on hemodynamic parameters than spinal-epidural anesthesia. This hemodynamic instability with spinal-epidural anesthesia is mainly induced by the sympathetic block [4–6]. For patients with severe aortic stenosis, it is important to maintain afterload in order to preserve coronary artery blood flow [7]. Otherwise, the vasodilating effects of anesthesia decrease afterload and cause hypotension. As a result, increased pressure gradient between the left ventricle and aorta may aggravate the myocardial oxygen demand-supply balance. We assessed afterload by evaluating SVRI. And SVRI had been stable during surgery.

Sedation was obtained with continuous administration of DEX in this patient because she had a concomitant psychiatric disorder and was expected to be unable to remain immobile during surgery [8]. DEX has been reported to induce hypercapnia [9]. In this study, however, although hypercapnia occurred after administration of intravenous 1.0 and 2.0 μg/kg DEX over a 2-min period, hypercapnia did not occur after administration of 0.25 and 0.5 μg/kg DEX. Otherwise, this case was administered DEX at 0.4–0.7 μg/kg/h without a loading dose, and the total DEX dose was within 0.6 μg/kg during the operation. Chang et al. reported that DEX produced less respiratory depression than propofol and midazolam in a sevoflurane-anesthetized rabbit CO_2_ inhalation model [10]. Accordingly, DEX was used to avoid hypercapnia due to respiratory depression because the patient had pulmonary hypertension. Alternatively, low-dose administration of DEX has been reported to elicit obstructive apnea [11]; moreover, fentanyl can lead to hypercapnia. In this case, however, DEX and fentanyl did not lead to hypercapnia (Table 1), and we believed that an additional local block would be useful. This case was sedated well, and hypercapnia was avoided. Hypotension is the primary significant side effect of DEX, which may be mitigated by avoiding a loading dose or initiating a slow infusion rate. In this patient, hemodynamic stability was a priority over her psychiatric disorder, so the DEX infusion was started without a loading dose [12]. Although continuous administration of DEX may decrease heart rate [13], this effect was considered to be rather advantageous for maintaining hemodynamics because she had concomitant atrial fibrillation and aortic stenosis. Lønnebakken et al. reported that a low SVI is associated with an increased cardiovascular mortality and high HR. The ACC/AHA guideline recommends avoiding tachycardia and hypotension in patients with coexisting aortic stenosis, so we maintained HR, SVI, and SVRI using the FloTrac/Vigileo system during the perioperative period [14]. She had anemia, so we monitored not only SVI but ScvO_2_ to assess oxygen supply/demand balance. We used additional 25 μg fentanyl four times when operative pain stimulation extended to the acetabuli, because the acetabuli received innervation from not only the femoral nerve but also the sciatic nerve. Although fentanyl was effective in controlling pain in this case, we believed that combining it with a sciatic nerve block would also be useful.

## Conclusions

Femoral nerve block with DEX was performed for noncardiac surgery in a patient with severe aortic stenosis. Her hemodynamics remained stable throughout the perioperative period, and there were no complications. In this case, anesthesia using a nerve block with DEX under hemodynamic monitoring contributed to the safety of noncardiac surgery in a patient with severe cardiac disease under conservative treatment during the perioperative period.

## Abbreviations

AHA/ACC: Association/American College of Cardiology
CI: Cardiac index
CVP: Central venous pressure
DEX: Dexmedetomidine
FNB: Femoral nerve block
HR: Heart rate
LFCNB: Lateral femoral cutaneous nerve block
MAP: Mean arterial pressure
OAA/S: Observer’s Assessment of Alertness/Sedation
ORIF: Open reduction and internal fixation
ScvO_2_: Central venous saturation
SVI: Stroke volume index
SVRI: Systemic vascular resistance index
